# Retraction Note: The risk factors of intracranial infection in patients with intracerebral hemorrhage undergone hematoma puncture: what should we care

**DOI:** 10.1186/s12879-023-08841-5

**Published:** 2023-11-28

**Authors:** Haijing Han, Yu Li, Li Liu, Ningning Liu, Ying Wang, Min Zhang

**Affiliations:** Department of Nursing, Xi’an International Medical Center Hospital, East of Xitai Road, High-tech Zone, Xi’an, 329000 Shaanxi Province China

**Retraction Note: BMC Infectious Diseases (2020) 20:949**

10.1186/s12879-020-05630-2

The Editor has retracted this article because the authors have been unable to provide documents confirming that ethics approval was obtained for the study. None of the authors have responded to correspondence from the Editor about this retraction.

